# Pioneer colonizers: Bacteria that alter the chicken intestinal morphology and development of the microbiota

**DOI:** 10.3389/fphys.2023.1139321

**Published:** 2023-03-29

**Authors:** Margie D. Lee, Adriana A. Pedroso, Brett Lumpkins, Youngjae Cho, John J. Maurer

**Affiliations:** ^1^ Virginia Maryland College of Veterinary Medicine, Virginia Tech, Blacksburg, VA, United States; ^2^ Poultry Diagnostic and Research Center, College of Veterinary Medicine, University of Georgia, Athens, GA, United States; ^3^ Department of Poultry Science, College of Agricultural and Environmental Sciences, University of Georgia, Athens, GA, United States; ^4^ Department of Animal and Poultry Sciences, College of Agriculture and Life Sciences, Virginia Tech, Blacksburg, VA, United States

**Keywords:** microbiome, performance, feed efficiency, intestinal development, anaerobes

## Abstract

Microbes commonly administered to chickens facilitate development of a beneficial microbiome that improves gut function, feed conversion and reduces pathogen colonization. Competitive exclusion products, derived from the cecal contents of hens and shown to reduce *Salmonella* colonization in chicks, possess important pioneer-colonizing bacteria needed for proper intestinal development and animal growth. We hypothesized that inoculation of these pioneer-colonizing bacteria to day of hatch chicks would enhance the development of their intestinal anatomy and microbiome. A competitive exclusion product was administered to broiler chickens, in their drinking water, at day of hatch, and its impact on intestinal morphometrics, intestinal microbiome, and production parameters, was assessed relative to a control, no treatment group. 16S rRNA gene, terminal restriction fragment length polymorphism (T-RFLP) was used to assess ileal community composition. The competitive exclusion product, administered on day of hatch, increased villus height, villus height/width ratio and goblet cell production ∼1.25-fold and expression of enterocyte sugar transporters 1.25 to 1.5-fold in chickens at 3 days of age, compared to the control group. As a next step, chicks were inoculated with a defined formulation, containing *Bacteroidia* and *Clostridia* representing pioneer-colonizing bacteria of the two major bacterial phyla present in the competitive exclusion product. The defined formulation, containing both groups of bacteria, were shown, dependent on age, to improve villus height (jejunum: 1.14 to 1.46-fold; ileum: 1.17-fold), goblet cell numbers (ileum 1.32 to 2.51-fold), and feed efficiency (1.18-fold, day 1) while decreasing *Lactobacillus* ileal abundance by one-third to half in birds at 16 and 42 days of age, respectively; compared to the phosphate buffered saline treatment group*.* Therefore, specific probiotic formulations containing pioneer colonizing species can provide benefits in intestinal development, feed efficiency and body weight gain.

## Introduction

The microbiome has been shown to serve as an effective barrier to pathogen colonization or pathogenic behavior in numerous examples while the mechanisms underlying pathogen exclusion remains elusive (Nurmi and Rantala, 1973; Berg, 1980; Faure et al., 1984). Approximately 50 years ago, Nurmi demonstrated that chicks seeded with the cecal microbiome from adult birds were resistant to *Salmonella* colonization (Nurmi and Rantala, 1973) and termed the phenomena “competitive exclusion”. Since this discovery, numerous groups have investigated single or multiple microbial species as probiotics to replace the effectiveness of growth-promoting antibiotics (Vuong et al., 2016) or suppress other harmful microorganisms (Fukata et al., 1991; Hofacre et al., 1998a). However, no defined consortium has been quite as effective at pathogen exclusion as Nurmi’s approach using the cecal microbiome. Competitive exclusion has since been commercialized; amplifying cecal bacteria from an original seed stock and distributing lyophilized cultures with >5 log10 *Salmonella* reduction to customers, marketed as a *Salmonella* exclusion product for poultry (Lee et al., 2023). Hofacre et al. demonstrated that administration of this competitive exclusion product could also reduce the severity of necrotic enteritis in poultry (Hofacre et al., 1998a). The study, which was replicated in 2019 with conditions intended to increase the severity of disease (Hofacre et al., 2019), illustrated an important new concept in disease control. The chicks were given one dose at day of hatch, then challenged with three sequential high oral doses of an avian pathogenic *C. perfringens* isolate 3 weeks later. The findings suggested a paradigm shift because the principle of competitive exclusion was inadequate to explain the chicks’ resistance to repeated doses of a billion pathogenic, toxigenic *Clostridium perfringens* cells administered orally for 3 days in a row 3 weeks after receiving the intestinal bioproducts. In fact, a subsequent study showed that one dose of the intestinal bioproduct, Aviguard^®^, performed as well as continuously feeding bacitracin or virginiamycin to prevent necrotic enteritis (Hofacre et al., 1998b). These findings indicated that competitive exclusion products could alter the intestinal environment leading to greater resistance to enteropathogens.

Understanding the microbiome, a consortium of microbes found in and on animal and plant species, offers a new perspective on eukaryotic development. An understanding of the nutritional and physiological contributions of the monogastric vertebrate intestinal microbiome is still emerging. The role of the microbiome in disease, physiology or development is often inferred from studies using “germ-free” subjects *versus* “conventionally-raised” litter mates. Monogastrics can be raised “germ-free” (gnotobiotic) (Pleasants, 1959; Meyer et al., 1964) however, gnotobiotic mice, pigs and rats exhibit reduced growth and weight gain compared to conventionally raised litter mates (Dymsza et al., 1965; Waxler and Drees, 1972; Al-Asmakh and Zadjali, 2015). Gnotobiotic animals are also more susceptible to enteric infections which makes them an excellent model for studying enteric pathogens (Sprinz et al., 1961; Eaton et al., 2008; Reeves et al., 2012). Augmentation with certain bacterial species has been shown have a profound effect on the intestinal physiology, growth, and disease resistance of gnotobiotic animals (Dymsza et al., 1965; Shirkey et al., 2006; Mahowald et al., 2009; Cheled-Shoval et al., 2014; Greig et al., 2018; Yin et al., 2022). Furthermore, the microbiome composition can have a profound impact on animal weight gain as evident when gnotobiotic mice receive fecal transplants from obese mice (Turnbaugh et al., 2006).

In comparisons with other germ-free animal models, chickens possessing an intestinal microbiota were believed to be at a growth disadvantage except when grown on vitamin-deficient diets (Coates et al., 1968) or those with high fiber/low metabolizable energy (Muramatsu et al., 1991). Gnotobiotic-chickens produce fewer goblet cells, more sulfated mucins (Cheled-Shoval et al., 2014), shorter villi, and lower crypt depth, compared to conventionally-raised birds (Cowieson, 2022). However, weight gain and feed conversion improved when chickens were raised with antimicrobial-amended feed but this was not observed in germ free animals (Lev and Forbes, 1959). Furthermore, it was most pronounced in birds raised in heavily contaminated environments indicating that the growth disadvantage was likely the result of pathogens. Therefore, in commercial poultry production, antibiotics such as virginiamycin and bacitracin improved growth performance on farms with high stocking density or poor hygienic conditions or practices (Eyssen and de Somer, 1963). Because this improvement was believed to be due to the suppression of pathogenic intestinal bacterial species such as *C. perfringens*, it led to the widespread use of antibiotics, in the U.S., as a prophylactic to prevent necrotic enteritis in poultry. This practice has been in decline since the European ban of growth-promoting antibiotics (Casewell et al., 2003) and the movement in the U.S. towards antibiotic free production (Diaz-Sanchez et al., 2015).

The maternal intestinal microbiome is an important source of organisms for the progeny and many studies have shown that the initial microbiome seeding is crucial for health of the progeny (Neu and Rushing, 2011; Koleva et al., 2015; Chen et al., 2018; Kubasova et al., 2019a; Klein-Jöbstl et al., 2019; Treichel et al., 2019; Yu et al., 2019). The modern poultry production system, in order to increase productivity and reduce disease transmission, eliminated the physical presence of the progenitors during the incubation and hatching process thereby interrupting the transfer of bacteria from hen to chick. As a result, newly hatched chicks do not have access to a diverse maternal microbiome are easily seeded with environmental microbes (Pedroso et al., 2015) and these organisms would not be expected to provide beneficial effects in early intestinal development or pathogen exclusion. Yet, there is a consistent and predictable microbial succession within the chicken intestine, beginning with oxygen-consuming streptococci and γ-proteobacteria at 4 days of age, followed by their displacement with the obligate anaerobic *Clostridia* (Lu et al., 2003; Jurburg et al., 2019). However, if chicks are presented early with cecal microbiome, they develop a stable community resistant to pathogen colonization (Ramírez et al., 2020). Because the microbiome affects animal physiology (Cheled-Shoval et al., 2014; Heath-Heckman et al., 2014; Kremer et al., 2014; McFall-Ngai, 2014), this would be especially evident in early intestinal development as the host responds to early pioneer colonizers (Shao et al., 2014; De Maesschalck et al., 2015; Gourbeyre et al., 2015).

The ecological concept of pioneer colonizers is well established and has been shown to be crucial in augmenting development of a functionally diverse intestinal microbiome. In 2005, Backhed et al. conceptualized the process by which pioneer colonizers coevolve with their animal hosts and influence the intestinal environment from a nutritional and anatomical standpoint (Backhed et al., 2005). Using gnotobiotic mice, they demonstrated that the developmental deficiencies associated with the absence of an intestinal microbiome, could be fully mitigated by administering a single *Bacteroides thetaiotaomicron* species. Subsequent studies illustrated that stem cell differentiation was stimulated by bacterial metabolites from utilization of host intestinal mucin (Sommer and Backhed, 2013). These findings indicated that probiotic formulations, containing pioneer colonizers from the intestinal microbiota of mature chickens, may accelerate intestinal development and improve performance in newly hatched chicks.

In this study, we treated day-of-hatch broiler chickens with a competitive exclusion product and investigated the impact of the treatments on intestinal community structure, as measured by 16 S rRNA gene terminal restriction fragment length polymorphism (T-RFLP), intestinal morphometrics (villus height, villus height to width ratio, goblet cell numbers), body weight gain and feed conversion ratios; compared to the no treatment, control group. As a second step, we selected specific species from chicken ceca, that represent the two dominant phyla in the competitive exclusion product Aviguard^®^ (Lee et al., 2023), and administered different formulations, consisting of these cecal organism, or PBS to newly hatched chicks in order to determine their effects on production performance, intestinal physiology and changes to the intestinal microbiome, relative to the PBS treatment group. Similar morphometric improvements to chicken gut function could be obtained with a two to five species, probiotic cocktail, consisting of obligate anaerobic pioneer colonizers, than a competitive exclusion product that consists of 20–50 distinct genera (Lee et al., 2023).

## Materials and methods

### 16S rRNA gene analysis of intestinal communities from chickens receiving a competitive exclusion product, probiotic formulation, or PBS

In order to recover bacteria from the commercial competitive exclusion product, the bacterial cells were rehydrated by incubation for 10 min in saline solution and recovered by centrifugation. Chicken intestines were collected from the various treatments and time points outlined below. The bacterial fraction was recovered from the intestinal contents through multiple rounds of differential centrifugation as described previously (Apajalahti et al., 1998; Lu et al., 2003). DNA was extracted using Mo Bio kit (Mo Bio Laboratories Inc., Solana Beach, CA), beating cell suspensions at 6,000 rpm for 20 min (Lu et al., 2008). The bacterial communities were assayed by 16 S rRNA terminal restriction fragment length polymorphism (T-RFLP) analysis, using a sequence-based database, as previously described (Lu et al., 2006; Lu et al., 2008). universal 16S rRNA primers 8F labeled with 5′FAM (carboxyfluorescein-N-hydroxysuccinimide ester-dimethyl sulfoxide) and unlabeled 1429R were used to amplify community DNA (Lu et al., 2008). Three separate 18-cycle PCR reactions were performed for each DNA sample and pooled for T-RFLP analysis. No DNA template was included with PCR, as a negative control. No amplicons were ever observed for this negative control. Amplicons were digested with restriction enzyme *Hae*III (New England BioLabs; Ipswich, MA) and analyzed by electrophoresis on ABI PRISM 310 DNA sequencer (PE Biosystems; Foster City, CA). For each sample, only peak areas and peak heights over a threshold of 50 units, above background were analyzed by manually aligning fragments to size standards; and only DNA fragments between 35 and 525 bp were examined. T-RFL peaks were identified by comparison to a 16S rRNA gene database, of Insilco *Hae*III patterns, from previously published clone libraries (Lu et al., 2003).

The relative abundance of bacterial species or phylotypes detected by T-RFLP was determined by calculating the ratio between the areas of each peak and the total areas of all peaks within one sample (Lukow et al., 2000); mean ratios of three analyses were converted to percentages. The Shannon diversity information index (Shannon and Wiener, 1963) was used to evaluate the diversity of the bacterial communities. The diversity indices were analyzed using analysis of variance (SAS, 2008) to determine differences between the intestinal communities from birds given Aviguard^®^ or nothing (no treatment control group).

### Isolation of pioneer colonizing bacteria from the chicken intestine

Aviguard^®^, consists predominantly of obligate anaerobes (Pedroso et al., 2015; Lee et al., 2023), belonging to *Clostridia* and *Bacteroidia* orders and because it improved intestinal morphometrics in young birds, we sought to isolate and identify pioneer colonizing species that could supplant this competitive exclusion product. Anaerobic, pioneer colonizing bacterial were isolated from the ceca of commercial broiler chicken carcasses obtained from a local processing plant. The cecal contents of three chickens were squeezed into pre-reduced serum bottles and serially diluted with 20 ml of phosphate-buffered saline (PBS) in within an anaerobic chamber containing 90% N_2_ and 10% H_2_. The suspensions were plated on rumen fluid-glucose-cellobiose plus peptone (RGCAP)-10, RGCAP-30, 10% modified rumen fluid medium (M98-5), and rich medium (RM) (ATCC Medium 1,341; 20 g glucose, 10 g yeast extract, 2 g K_2_HPO_4_, 15 g agar per 1L dH2O) agar (Kelley, 1983), and incubated under 95% N_2_ and 5% H_2_ for 5 days at 41°C. Isolated colonies were characterized by 16 S rDNA sequencing as previously described (Lu et al., 2003). Selected isolates of *Escherichia coli*, *Parabacteroides distasonis*, *Bacteroides salyersiae*, *Phocaeicola dorei* and *Romboutsia lituseburensis* ATCC 25759 were grown on RGCAP-10 agar under anaerobic conditions (80% N_2_, 10% CO_2_ and 10% H_2_) for 5 days. Colonies were harvested and resuspended in pre-reduced saline solution to reach the concentration of 10^9^ CFU/ml.

Obligate anaerobes were isolated by culture and identified by 16S rRNA amplicon sequencing. Subculture yielded obligate anaerobes belonging to the order *Bacteroidia* which were identified as *P. distasonis*, *B. salyersiae*, and *P. dorei* (formerly, *Bacteroides dorei*). Partial sequence of their genomes revealed polysaccharide utilization loci and associated glycosyl hydrolases (Grondin et al., 2017) characteristic of the *Bacteroidia.* DNA sequence of these genes, 16S rRNA, and other housekeeping genes confirmed their identity to the species level (BLAST scores: ≥98% nucleotide identity; 100% coverage) (Table 1). The *Bacteroidia* genomes exhibited several annotated genes for acetate and propionate metabolism. *Bacteroides salyersiae* and *P. dorei* also possessed genes annotated as phosphotransbutyrylase and butyrate kinase, responsible for butyrate production. These genes were absent in a search of the isolated *P. distasonis’* genome as well as a search of published, annotated *P. distasonis* genomes, including a specified BLAST search, at the amino acid level. While these *Bacteroidia* contained core carbohydrate-active enzymes (CAZymes), there were differences in the distribution of other CAZymes among these isolates. Because of the variances in carbohydrate and fermentation metabolism, it was decided to include multiple species as part of a *Bacteroidia* cocktail to administer to birds.

**TABLE 1:** Cecal bacteria used to formulate probiotics described in this study.

Group	Species	Features	Identity^i^	Source
*Bacteroidia*	*Parabacteroides distasonis*	*susC,D* polysaccharide utilization loci^b^, associated glycosyl hydrolases (11), core saccharidases^c^ and additional enzymes annotated as: arabinogalactan endo-1,4-β-galactosidase, α−mannosidase, α-glucosidase, *ß*-glucanase 2), α-1,6-mannanase, mannan endo-1,4-β-mannosidase; conjugative transposon; respiratory hydrogenases and cytochromes; propionate metabolism^d^; acetyl-CoA hydrolase	16S, 23 S rRNA, *gyrA*, *susC,D* loci, Supplemental Files^e^	Chicken cecum^f^; RGCAP-30 medium^g^
	*Bacteroides salyersiae*	*susC,D* polysaccharide utilization loci^b^, associated glycosyl hydrolases, core saccharidases^c^ and additional enzymes annotated as: arabinosidase, α-glucosidase, rhamnogalacturonan lyase, α−mannosidase pectate lyase, α-1,6-mannanase, α-glucosidase; propionate metabolism^d^ including propionyl-CoA carboxylase, butyrate metabolism^h^	23 S rRNA, *susC,D* loci, ATP synthase subunit, Supplemental Files^e^	Chicken cecum^f^; RGCAP-30 medium^g^
	*Phocaeicola dorei*	*susC,D* polysaccharide utilization loci^b^, associated glycosyl hydrolases, core saccharidases^c^ and additional enzymes annotated as: arabinosidase, arabinogalactan endo-1,4-β-galactosidase, rhamnogalacturonan lyase, pectate lyase; propionate metabolism^d^ including propionyl-CoA carboxylase, butyrate metabolism^h^, acetyl-CoA hydrolase	23 S rRNA, *susC,D* loci, ATP synthase subunit, Supplemental Files^e^	Chicken cecum^f^; RGCAP-30 medium^g^
*Clostridia*	*Romboutsia lituseburensis* ^a^	Glycosyl or glycoside hydrolases (17) including: α or *ß*-glucosidase, *ß*-galactosidase, α-mannosidase; butyrate metabolism^h^, lactate dehydrogenase, formate dehydrogenase, acetyl-CoA decarbonylase/synthase complex, and Fe-Fe hydrogenases; ethanolamine utilization; vitamin B12 synthesis; flagella/motility; sporulation	NA	ATCC 25759
γ-Proteobacteria	*Escherichia coli*	Aerobic/anaerobic respiration including hydrogenases associated with H_2_ consumption; enzymes and transporters associated with di- and mono-saccharide metabolism	16S, 23 S rRNA, *gyrA*, respiratory hydrogenases *hyfJ*, *hybF*, nitrate reductase *napF*; Supplemental Files^e^	Chicken cecum^f^

^a^ Features inferred from the annotated genome for *Romboutsia lituseburensis* DSM, 297 (NCBI, RefSeq: NZ_FNGW00000000.1).

^b^ *susC,D*: signature polysaccharide transport proteins commonly associated with polysaccharide utilization in *Bacteroidia* (Grondin et al., 2017). Other genes associated with these loci include: glycosyl hydrolases and regulatory genes: membrane sensor, alternate sigma factor and anti-sigma factor. These same ancillary genes have also been reported as part of polysaccharide utilization loci in other *Bacteroidia* genomes (Grondin et al., 2017).

^c^ Fucosidase, glucoamylase, α-amylase, neopullulanase (*susA*), sialidase, arabinofuranosidase, polygalacturonase, *ß*-galactosidase, *ß*-glucosidase, α-1,2 mannosidase, *ß*-mannosidase, *ß*-hexosaminidase, α-rhamnosidase, maltodextrin glucosidase, *ß*-xylosidase.

^d^ Methylmalonyl-CoA, mutase, methylmalonyl-CoA decarboxylase, methylmalonyl-CoA, epimerase.

^e^ “Supplemental Excel Files: *Clostridia Bacteroidia* Probiotic Formulation Sample one and 2.

^f^ Bacteria were isolated from cecal contents of chicken carcasses collected at a poultry processing plant.

^g^ Reference: (Kelley, 1983).

^h^ Phosphotransbutyrylase, butyrate kinase.

^i^ Identity confirmed at nucleotide level by BLAST (Altschul et al., 1990) with 100% query coverage and 98%–100% identity.

As *R. lituseburensis* was an abundant phylotype in birds fed the competitive exclusion product Aviguard^®^ or other feed additives (Lu et al., 2008), an *R. lituseburensis* isolate was purchased from the American Type Culture Collection (ATCC 25759) to be included in this study. In addition, an *E. coli* isolated from the chicken intestinal samples, served as a γ-proteobacteria pioneer for establishing the anaerobic environment needed for seeding chicks with obligate anaerobes (Espey, 2013).

Pools of isolates were created by mixing equal volumes of suspensions. Three pools of probiotic cultures were prepared for administration to day of hatch chicks and consisted of the following formulations; probiotic cocktail 1: *P. distasonis*, *B. salyersiae*, and *P. dorei*, and *E. coli*; probiotic cocktail 2: *R. lituseburensis* and *E. coli*; and probiotic cocktail three containing *P. distasonis*, *B. salyersiae*, *P. dorei*, *R. lituseburensis and E. coli*. Glycerol (15%) was added to aliquots of each probiotic formulation and stored at −80°C.

### Birds treated with competitive exclusion product

For assessment of the effects of the commercial competitive exclusion product (Aviguard^®^, Lallemand, Montreal Canada), 120 one-day-old commercial Ross-Cobb hybrid broiler chicks were raised in two groups of 60 on sawdust bedding. Both groups were fed a commercial corn-soy bean meal diet devoid of antimicrobials. Chicks in one group were administered the commercial competitive exclusion product, Aviguard^®^ in their drinking water on the day of hatch, as per the manufacturer’s instructions, while the other group just received standard drinking water, no product. Birds were sacrificed, by cervical dislocation, at 3, 7, 14, 21, 28, and 49 days of age and intestines were collected. The ileal contents were collected and processed as previously described for 16S rRNA gene TRFLP analysis. Intestinal morphometrics and glucose transporter gene expression were performed on intestines collected from birds at 3 days of age, as described below.

### Birds treated with probiotic cocktails

Eight hundred and 40 day of hatch chicks (Cobb 500) were divided into four treatments of three replications each containing 70 chicks. Chicks were orally inoculated with 50 µl of 1 × 10^8^ *Bacteroidia* cocktail (*P. distasonis*, *B. salyersiae*, and *P. dorei*) with *E. coli, R. lituseburensis* with *E. coli,* and *Bacteroidia* cocktail with *R. lituseburensis and E. coli.* The control group received 50 μl sterile PBS. Chickens were fed a corn-soy bean meal diet free of antimicrobials (Table 1). Birds were sacrificed by cervical dislocation 3 hours following oral administration with probiotic formulation or PBS and at days 1, 2, 3, 7, 16 and 42 days and intestines were collected.

### Intestinal histology and morphometrics

Following inoculation with the competitive exclusion product Aviguard^®^ (*n* = 60), probiotic formulation (3 different formulations, 210 birds per treatment) or PBS (*n* = 210), chickens were sacrificed at 3 hours after inoculation, or at time points described above and the small intestines were collected. A no treatment group (*n* = 60) was included with the competitive exclusion trial. The middle portion of the jejunum and ileum from 4 birds per experimental unit were excised, fixed in 10% formalin, embedded in paraffin and cut in five um thick sections. Three intact, well-oriented villi were selected in eight replicates for each intestinal cross section, totaling 24 villus height and width measurements for each intestinal sample and 288 measurements per treatment. In addition, intestinal sections were stained using Mayer’s Mucicarmine (Val-Bernal et al., 1999) and the number of goblet cells counted. Morphological indices were determined using a light microscope and a ×16 magnification lens. Images were analysis using the Image-Pro Plus Version 3.0 software (Media Cybernetics, Silver spring, MD). Expression of glucose transporters were measured by reverse-transcriptase (RT) qPCR according to method described by Gilbert et al. (2007).

The jejunum and ileum from four animals per treatment group (three probiotic cocktails and PBS control) were collected, measured, flushed using deionized water, and the empty weight recorded. Relative intestinal weight (grams/kg of body weight) and relative intestinal lengths (mm/kg of body weight) were determined.

### Animal performance

Body weight and feed intake were recorded and body weight gain and gain: feed were calculated. At the occurrence of mortality, feed intake was adjusted based on bird days on feed. At 42 days of age, fifteen chickens per pen were randomly selected and wing-banded and fasted overnight. Birds were weighed individually, slaughtered, eviscerated, and carcasses were chilled for 12 h. The yield was obtained for the entire carcass, and parts.

### Whole genome sequencing and genomic analyses of *Parabacteroides distasonis, Bacteroides salyersiae, Phocaeicola dorei, Escherichia coli* and *Romboutsia lituseburensis* probiotic cocktail

Two samples were thawed on ice, centrifuged at 10,000xg at 4°C for 15 min, and DNA was extracted from the bacterial pellet using Promega Wizard^®^ Genomic DNA extraction kit (Madison, WI), with an added lysozyme treatment, as described by the manufacturer. DNA was submitted to Georgia Genomics and Bioinformatics Core for sequencing using Illumina sequencing (San Diego, CA). FastQC and FastQ/A were used to clean raw sequence reads of adapters and low-quality sequences (Patel and Jain, 2012; Afgan et al., 2018). SPADES sequence alignment tool was used to assemble processed pair-end Illumina reads (Bankevich et al., 2012). Assembled sequence files were uploaded and annotated in the Rapid Annotation using Subsystem Technology (RAST) (Aziz et al., 2008). Species identity of individual contigs (≥17 kb) was determined by Basic Local Alignment Search Tool (BLAST) (Altschul et al., 1990) at nucleotide level (≥98% identity). Identity of species, within this probiotic formulation, was confirmed by BLAST for sequences annotated as: “small ribosomal subunit RNA” (16 S rRNA); “large ribosomal subunit RNA (23S rRNA); gene annotated as “SusC”, “SusD”, polysaccharide utilization genes commonly present in the *Bacteroidia* (Grondin et al., 2017); or housekeeping genes listed in Table 1 (≥98% identity, 100% coverage). The *Bacteroidia* are adept at metabolizing complex carbohydrates, whether its indigestible fiber from the animal’s diet or mucin, and producing volatile fatty acid from said metabolism for its host. As multiple *Bacteroidia* species were identified, a more detailed genomic analysis was performed to determine which isolates to include in the probiotic formulation that had the broadest repertoire of carbohydrate metabolism. Carbohydrate-active enzymes (CAZymes) were identified among the annotated sequences through a word search for genes annotated as “Sus”, “glycosyl hydrolases”, “amylase”, “pullanase” or “idase”; and species identity and enzyme confirmation was determined by BLAST at the nucleotide and amino acid level, respectively. CAZymes identified had motifs consistent with these enzymes at the amino acid level. In *Bacteroidia*, CAZymes are often associated with Polysaccharide Utilization Loci (PUL) denoted by polysaccharide transporters Sus (Grondin et al., 2017). Several loci were identified with Sus minus any genes annotated as some CAZyme. Genes annotated as “hypothetical protein” were identified as CAZyme *via* BLASTX search of annotated bacterial genomes. Fermentation enzymes were identified by similar word search of gene annotations for enzymes listed in Table 1. Their identity and species assignment were determined by BLAST search at the amino acid and nucleotide level, respectively.

### Statistical analysis

Performance and intestinal measurements were subjected to Analysis of Variance (ANOVA) procedure for completely randomized design using the general linear model procedure of SAS (SAS, 2008). Statistical significance of differences among treatments was assessed using the least significant difference test (Steel and Torrie, 1980). A probability level of *p* < 0.05 was used to determine statistical significance. The Standard Error of Measure (SEM) was calculated from the standard deviation of all values divided by the square-root of the sample size.

## Results

### Competitive exclusion product improves intestinal morphology and enterocyte function

**FIGURE 1:**
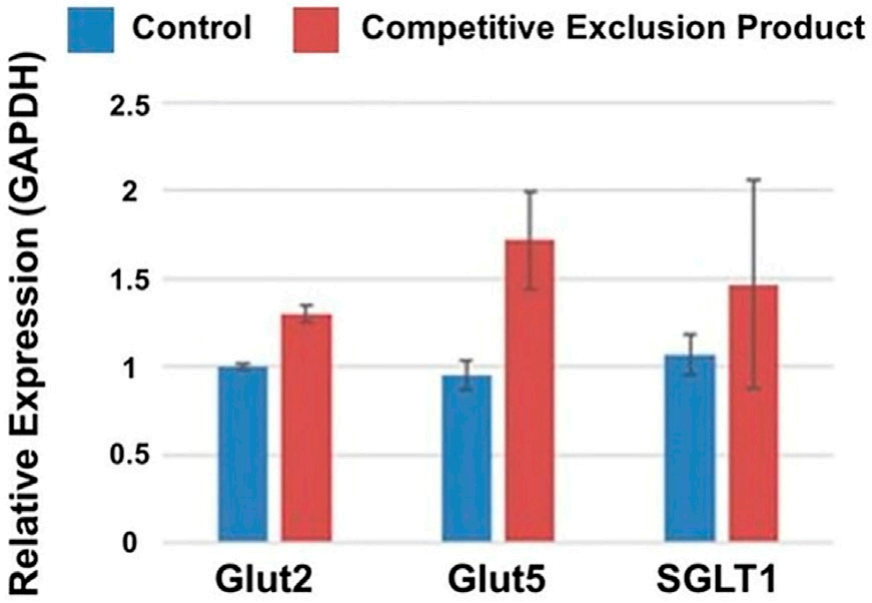
Relative expression of glucose transporters Glut2, Glut5 and SGLT1 (*p* < 0.05) in the small intestine of 3 d old broiler chickens receiving a competitive exclusion product (Aviguard^®^) or no treatment (control) on the day of hatch. Relative gene expression was determined using the 2^−ΔΔCT^ method. GAPDH was used to normalize gene expression for targeted genes.

While competitive exclusion products have been shown effective at pathogen exclusion, can this microbial consortium, of chicken intestinal origin, also effectively modulate intestinal morphology and function? To address this question, birds were either administered the competitive exclusion product, Aviguard^®^ at day of hatch, in their drinking water or not (untreated, control group). Administration of Aviguard^®^ improved intestinal morphology, increasing villus height, height/width ratio; and percentage of goblet cells per villus 1.26 to 1.36-fold, *p* < 0.05 (Table 2), compared to untreated birds. Furthermore, increased expression 1.25 to 1.5-fold of the enterocyte transporters, GLUT2, GLUT5, and SGLT1 was exhibited in the ileum compared to control group in 3-day-old broiler chickens (Figure 1; *p* < 0.05).

**TABLE 2:** Ileal morphology of broiler chicks at 3 days of age that were administered an intestinal bioproduct Aviguard^®^.

	Villus height (μm)	Height/width ratio	Crypt area (μm^2^)	Mucosal layer width (μm)	Numbers of goblet cells per villus
Control	362.73^b^	2.58^b^	6,735.60^a^	141.94^a^	8.24^b^
Aviguard®	455.92^a^	3.52^a^	6,946.10^a^	155.53^a^	10.96^a^

Different superscripts within each column indicate significant differences, *p* < 0.05.

### Competitive exclusion product stabilizes community diversity and promotes clostridia abundance in the chicken ileum

**FIGURE 2:**
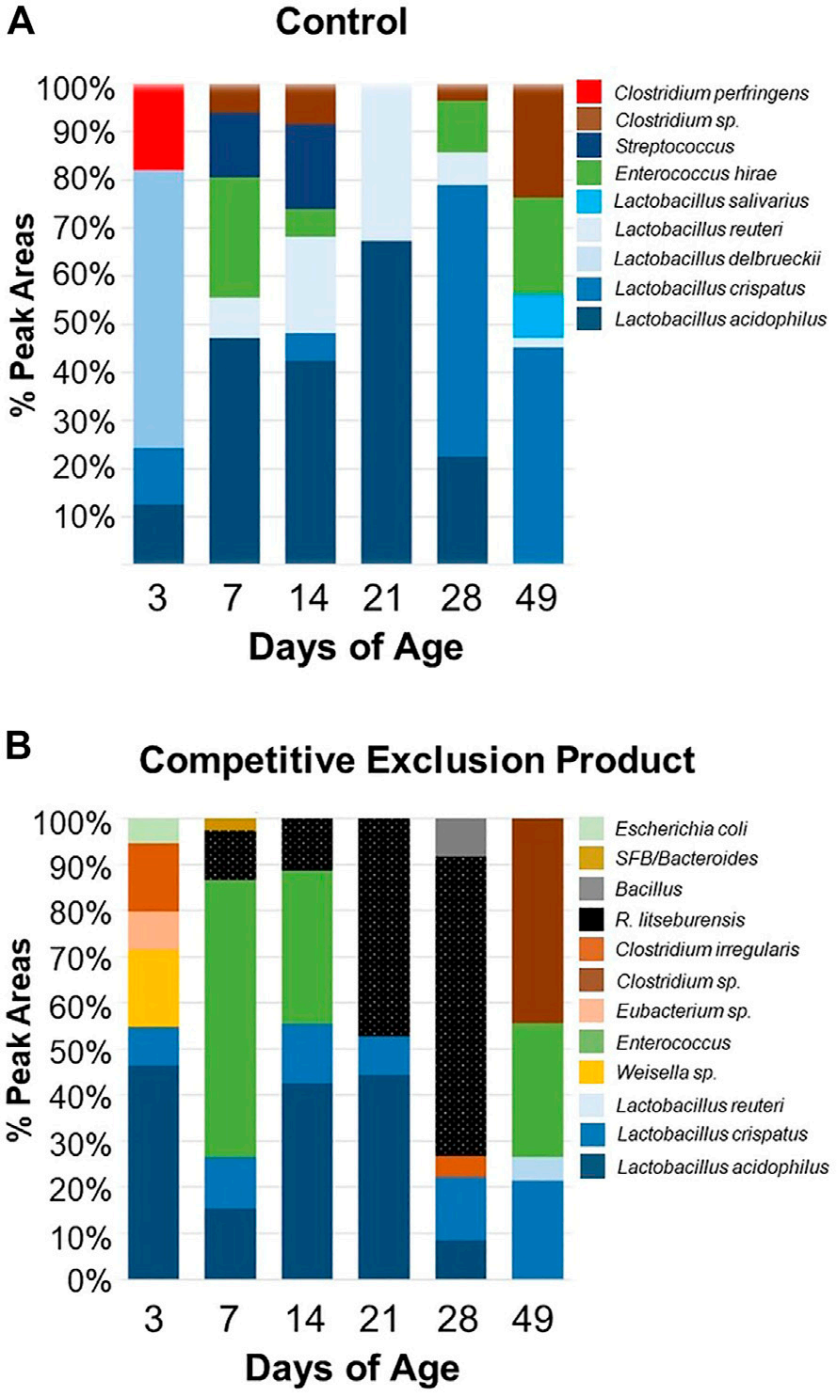
Composition of the ileal bacterial community of chicks administered Aviguard^®^ (panel **(B)**) or no treatment (control, Panel **(A)** on day of hatch as determined by 16S rRNA T-RFLP at 3, 7, 14, 21, 28 and 49 days of age.

**FIGURE 3:**
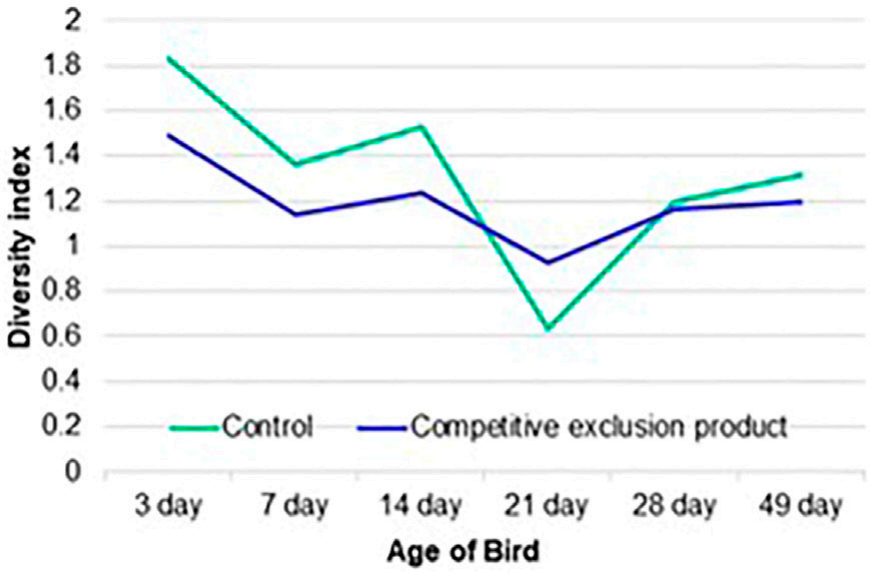
Shannon’s H diversity index of samples collected from the ileal bacterial community of chicken from the control (no treatment) or birds administered a competitive exclusion product (Aviguard^®^) at day of hatch. Samples were collected at 3, 7, 14, 21, 28 and 49 days of age.

Figure 2 and Figure 3 illustrate the composition and successions of bacteria in the microbiome in response to administration of Aviguard^®^ to birds at day of hatch. There were differences in the succession of bacterial phylotypes over the 49-day period between the control and birds administered the competitive exclusion product, Aviguard^®^ (Supplementary Figure S1). There were also significant differences in the distribution of phylotypes between the control and birds administered the competitive exclusion product, especially evident were differences in *Lactobacillus crispatus* and *R. lituseburensis* (*Clostridia*) abundance. This was most pronounced in birds at 21 days of age and older (Figure 2). *Lactobacillus* species were the most abundant group in untreated birds (Figure 2, Supplementary Figure S1), while the abundance of other species varied. However, administration of Aviguard^®^ produced an ileal bacterial community in which the *Clostridia* were abundant at 3 days of age while *Enterococcus* phylotypes represented 60% of total phylotypes on day 7 (Figure 2, Supplementary Figure S1). But SFB/*Bacteroides* phylotypes emerged with *R. lituseburensis* day 7 with *Romboutsia* becoming the most abundant ileal species by day 28 representing 70% of the total phylotypes for the treatment group. This observation suggested that *Bacteroides* may act as a pioneer colonizer in chicks enabling successional colonization of poultry anaerobic bacteria.

The Shannon diversity index indicated that age-related instability in the diversity of the ileal communities could be reduced by Aviguard^®^ (Figure 3). There were significant differences between the control and treatment groups at all ages analyzed (*p* < 0.05). At 21 days of age, there was a distinct reduction in diversity which was most pronounced for the control group. Aviguard^®^ administration lowered diversity but provided stability compared to the dramatic shifts in control birds.

### Pioneer colonizers promote intestinal function and growth performance

Newly hatched chicks inoculated with *R. lituseburensis and E. coli cocktail* had the greatest (15.8 vs 14.7 g; *p* < 0.05), body weight gain 24 h following its administration (Table 3), relative to the PBS control. Similarly, the feed: gain ratio was improved 1.18-fold relative to PBS control (1,408 vs. 1,198 kg/g; *p* < 0.05) in chicks receiving the *P. distasonis*, *B. salyersiae*, *P. dorei*, *E. coli* and *R. lituseburensis* cocktail. In addition, *R. lituseburensis* and *E. coli* cocktail or the *P. distasonis*, *B. salyersiae*, *P. dorei*, *E. coli* and *R. lituseburensis* cocktail produced a higher body weight gain at 16 days of age (438 or 421 vs. 411; *p* < 0.05). Probiotic cocktails consisting of *R. lituseburensis* and *E. coli* or *P. distasonis*, *B. salyersiae*, *P. dorei*, *E. coli* and *R. lituseburensis* reduced body weight gains by 4% at the end of the rearing period, compared to birds administered PBS or the *P. distasonis*, *B. salyersiae*, *P. dorei*, and *E. coli* cocktail. However, birds administered *R. lituseburensis* and *E. coli* cocktail had higher carcass yield (Table 4; 76.1% vs 73.7%, *p* < 0.05). No differences were observed on the legs, thighs, wings and breast yield for either probiotic formulations or PBS control therefore the weight gains were likely tied to changes in intestinal development.

**TABLE 3:** Body weight gain and feed efficiency of birds inoculated with *Parabacteroides distasonis*, *Bacteroides salyersiae*, *Phocaeicola dorei*, and *Escherichia coli* (Cocktail 1); *Romboutsia lituseburensis* and *E. coli* (Cocktail 2); *P. distasonis*, *B. salyersiae*, *P. dorei*, *E. coli* and *R. lituseburensis* (Cocktail 3); or PBS.

	Body weight gain g)						Gain: Feed (g/kg)					
Treatment	1 d	2 d	3 d	7 d	16 d	42 d	1 d	2 d	3 d	7 d	16 d	42 d
PBS	14.8^b^	13.9	16.5	101	411^b^	2551^a^	1189^c^	1,086	929	864	740	573
Cocktail 1	15.0^b^	13.4	16.7	103	429^a^ ^,^ ^b^	2527^a^	1225^b^ ^,^ ^c^	999	916	871	787	580
Cocktail 2	15.7^a^	13.1	16.7	105	438^a^	2448^b^	1372^a^ ^,^ ^b^	1,137	933	861	777	570
Cocktail 3	14.8^b^	13.6	16.4	103	421^a^	2427^b^	1408^a^	1,063	948	852	760	577
SEM	0.19	0.22	0.26	1.16	5.49	26.99	47.82	66.78	54.58	19.54	13.58	4.84

^a-c^ Means within a column and parameter with no common superscript differ significantly (*p* < 0.05).

**TABLE 4:** Carcass yield (%) of chickens at 42 d old that were administered *Parabacteroides distasonis*, *Bacteroides salyersiae*, *Phocaeicola dorei*, and *Escherichia coli* (Cocktail 1); *Romboutsia lituseburensis* and *E. coli* (Cocktail 2); *P. distasonis*, *B. salyersiae*, *P. dorei*, *E. coli* and *R. lituseburensis* (Cocktail 3); or PBS.

Treatment	Carcass	Legs	Thighs	Wings	Breast
PBS	73.7^b^	14.4	17.2	12.7	21.4
Cocktail 1	74.2^b^	14.5	18.2	11.8	21.4
Cocktail 2	76.1^a^	14.2	18.2	10.9	21.2
Cocktail 3	74.2^b^	14.0	17.7	11.3	21.0
SEM	0.49	0.14	0.36	0.62	0.61

^a,b^ Means within a column with no common superscript differ significantly (*p* < 0.05).

During the first week, changes in the intestinal development were observed in response to the probiotics administered to day of hatch chicks. Birds administered the *P. distasonis*, *B. salyersiae*, *P. dorei*, and *E. coli* cocktail had a higher relative jejunal weight, 1.28 to 1.44-fold increase, just 3 h following administration compared to chicks receiving *R. lituseburensis* and *E. coli* cocktail or the PBS control, respectively (Table 5). At 2 days of age, the group that received *P. distasonis*, *B. salyersiae*, *P. dorei*, and *E. coli* cocktail had a relative jejunal weight, ∼1.2-fold greater than the control or the other probiotic formulations. The relative weight of the jejunum was significantly decreased by 18% for birds administered *R. lituseburensis* and *E. coli* cocktail in comparison to the PBS control at 42 days (*p* < 0.05). There were no significant differences in jejunal length for either probiotic administration compared to the control. The probiotics also did not impact the relative weight or length of the ileum (Supplementary Table S2).

**TABLE 5:** Relative intestinal weight and length of the jejunum of chickens administered *Parabacteroides distasonis*, *Bacteroides salyersiae*, *Phocaeicola dorei*, and *Escherichia coli* (Cocktail 1); *Romboutsia lituseburensis* and *E. coli* (Cocktail 2); *P. distasonis*, *B. salyersiae*, *P. dorei*, *E. coli* and *R. lituseburensis* (Cocktail 3); or PBS.

	0 d	1 d	2 d	3 d	7 d	16 d	42 d
Treatment		Relative weight (g/kg of BW)					
PBS	10.0^b^	20.5	22.3^b^	27.6	29.3	22.9	9.8^a^
Cocktail 1	14.4^a^	21.9	26.6^a^	27.0	29.2	21.8	9.3^a^
Cocktail 2	9.8^b^	19.4	22.1^b^	29.5	31.1	22.4	8.0^b^
Cocktail 3	12.6^ab^	20.0	22.7^b^	25.6	28.3	20.2	8.9^ab^
SEM	0.93	1.12	1.09	1.42	1.15	0.86	0.43
Relative length (cm/kg of BW)							
PBS	318	321	303	309	181	89	27
Cocktail 1	375	342	316	306	188	83	28
Cocktail 2	336	308	315	311	193	83	28
Cocktail 3	339	322	321	293	179	84	27
SEM	17.05	14.04	11.22	11.55	6.42	2.98	1.32

^a-c^ Means within a column with no common superscript differ significantly (*p* < 0.05).

^a^ 3 h following administration of probiotic.

The *P. distasonis*, *B. salyersiae*, *P. dorei*, *E. coli* and *R. lituseburensis* cocktail induced longer jejunal villi just 3 h following administration (Table 6; Supplementary Figure S2), and continued to increase villus height at 7 and 16 days of age, compared to the PBS control (1.46, 1.14, and 1.15-fold increase, respectively; *p* < 0.05). However, the villus height was shorter in birds at 2 and 3 days of age, for *R. lituseburensis* and *E. coli* cocktail or *P. distasonis*, *B. salyersiae*, *P. dorei*, and *E. coli* cocktail compared to the PBS control (∼20% decrease; *p* < 0.05). By 42 days of age, there were no significant differences in jejunal villus height for either group. The probiotic formulations did not seem to elicit enhancement of villi height in the ileum as seen in the jejunum until birds were 42 days of age. At this time point, all three formulations increased villi height compared to the PBS control with *R. lituseburensis* and *E. coli* cocktail or *P. distasonis*, *B. salyersiae*, *P. dorei*, *E. coli* and *R. lituseburensis* having the most pronounced effect on villus height (1.39 and 1.16-fold increase, respectively; *p* < 0.05). At earlier time points, the probiotics appeared to reduce ileal villus height, compared to the control group, 3 h (*P. distasonis*, *B. salyersiae*, *P. dorei*, and *E. coli* cocktail; 40% decrease; *p* < 0.05) following probiotic administration; and at day 7, all three probiotic formulations reduced villus height ∼20% relative to the PBS control (*p* < 0.05).

**TABLE 6:** Jejunal and ileal villi height of chickens administered *Parabacteroides distasonis*, *Bacteroides salyersiae*, *Phocaeicola dorei*, and *Escherichia coli* (Cocktail 1); *Romboutsia lituseburensis* and *E. coli* (Cocktail 2); *P. distasonis*, *B. salyersiae*, *P. dorei*, *E. coli* and *R. lituseburensis* (Cocktail 3); or PBS.

	0 d	1 d	2 d	3 d	7 d	16 d	42 d
Treatment	Jejunum (µm)						
PBS	149^b^	205	246^ab^	287^a^	409^b^	580^b^	692
Cocktail 1	136^b^	218	198^c^	242^b^	422^b^	654^a^	708
Cocktail 2	124^b^	187	226^bc^	245^b^	441^ab^	645^a^	732
Cocktail 3	218^a^	221	264^a^	272^ab^	467^a^	668^a^	694
SEM	12.3	9.4	15.2	10.9	13.6	23.2	23.2
Ileum (µm)							
PBS	115^a^	183^ab^	182	223	260^a^	314	379^c^
Cocktail 1	72^b^	180^ab^	187	214	238^b^	320	423^b^
Cocktail 2	122^a^	168^b^	192	222	232^b^	304	529^a^
Cocktail 3	139^a^	190^a^	207	222	239^b^	311	443^a^
SEM	8.9	6.2	19.8	10.3	6.5	16.9	14.2

^a-c^ Means within a column with no common superscript differ significantly (*p* < 0.05).

^a^ 3 h following administration of probiotic.

The *P. distasonis*, *B. salyersiae*, *P. dorei*, *R. lituseburensis* and *E. coli* cocktail significantly increased 1.3 to 2.5-fold the number of goblet cells in the ileum in newly hatched chicks, just 3 h following its administration, and at day 2 in the ileum, respectively (Table 7; *p* < 0.05). The *P. distasonis*, *B. salyersiae*, *P. dorei*, and *E. coli* cocktail increased goblet cell numbers 1.5-fold at day 3 and the *R. lituseburensis* and *E. coli* cocktail improved goblet cells numbers at day 7 relative to the PBS control (*p* < 0.05). All probiotic formulations increased ∼1.5-fold goblet cells per villus at 42 days (Table 7), in the ileum, however, a significant decrease was observed in the proportion of goblet cells in the jejunum with probiotic formulations *R. lituseburensis* and *E. coli* (30% reduction, day 7), or *P. distasonis*, *B. salyersiae*, *P. dorei*, *R. lituseburensis* and *E. coli* cocktail (28% and 40% reductions on days 3 and 42, respectively) relative to PBS control (Table 7; *p* < 0.05).

**TABLE 7:** Goblet cells number per jejunum and ileal villus length of chickens inoculated *Parabacteroides distasonis*, *Bacteroides salyersiae*, *Phocaeicola dorei*, and *Escherichia coli* (Cocktail 1); *Romboutsia lituseburensis* and *E. coli* (Cocktail 2); *P. distasonis*, *B. salyersiae*, *P. dorei*, *E. coli* and *R. lituseburensis* (Cocktail 3); or PBS.

	0 d^+^	1 d	2 d	3 d		7 d	16 d	42 d
Treatment	Jejunum (number of goblet cells per villus length (μm))*							
PBS	0.1824 ± 0.0161	0.1251 ± 0.0139^a,b^	0.1108 ± 0.0201	0.0889 + 0.0105^a^		0.1048 ± 0.0094^a^	0.0796 ± 0.0087	0.2429 ± 0.0390^a^
Cocktail 1	0.1561 ± 0.0169	0.0969 ± 0.0077^a^	0.0888 ± 0.0137	0.0733 + 0.0095^a,b^		0.1187 ± 0.0092^a^	0.0893 ± 0.0097	0.1804 ± 0.0202^a,b^
Cocktail 2	0.2005 ± 0.0145	0.1941 ± 0.0269^b^	0.0941 ± 0.0053	0.0694 + 0.0145^a,b^		0.0723 ± 0.0059^b^	0.0777 ± 0.0071	0.1750 ± 0.0172^a,b^
Cocktail 3	0.1963 + 0.0096	0.1223 ± 0.0136^a,b^	0.0990 ± 0.0054	0.0638 + 0.0041^b^		0.0835 ± 0.0125^a,c^	0.0931 ± 0.0087	0.1443 ± 0.0032^b^

Treatment	Ileum (number of goblet cells per villus length (μm))*							
PBS	0.1918 + 0.0097^a^	0.1054 ± 0.0125	0.0707 ± 0.0071^a,b^	0.0810 + 0.0066^a^		0.1365 ± 0.0167^a,c^	0.1172 ± 0.0211^a,b^	0.1939 ± 0.0210
Cocktail 1	0.2024 + 0.0162^a,b^	0.1155 ± 0.0115	0.0553 ± 0.0105^a^	**0.1223 ± 0.0162** ^**b**^		0.1030 + 0.0135^a^	0.0775 ± 0.0076^a,b^	**0.2796 ± 0.0273** ^**a,c**^
Cocktail 2	0.2018 + 0.0081^a,b^	0.1083 ± 0.0142	0.0871 ± 0.0116^b^	0.0690 ± 0.0070^a,c^		**0.1512 + 0.0118** ^**b,c**^	0.1045 ± 0.0094^a,c^	**0.3387 ± 0.0175** ^**b,c,d**^
Cocktail 3	**0.2536 + 0.0270** ^**b**^	0.1060 ± 0.0129	**0.1777 ± 0.0209** ^**c**^	0.0988 + 0.0136^a,b,d^		0.1383 ± 0.0172^a,c^	0.0930 ± 0.0075^a,c^	**0.2901 ± 0.0256** ^**c,d**^

^a-d^ Means within a column and parameter with no common superscript differ significantly (*p* < 0.05). ^+^3 h following administration of probiotic. *As villus length increases with, goblet cell numbers per villus was normalized against villus length. **Bold** denote statistically significant increase in goblet cells in response to probiotic *versus* the control. Bold values significantly different (*p* < 0.05) from PBS control.

### *P. distasonis*, *Bacteroides salyersiae*, *P. dorei*, *Romboutsia lituseburensis and Escherichia coli* cocktail lower *Lactobacillus* abundance in the chicken ileum

**FIGURE 4:**
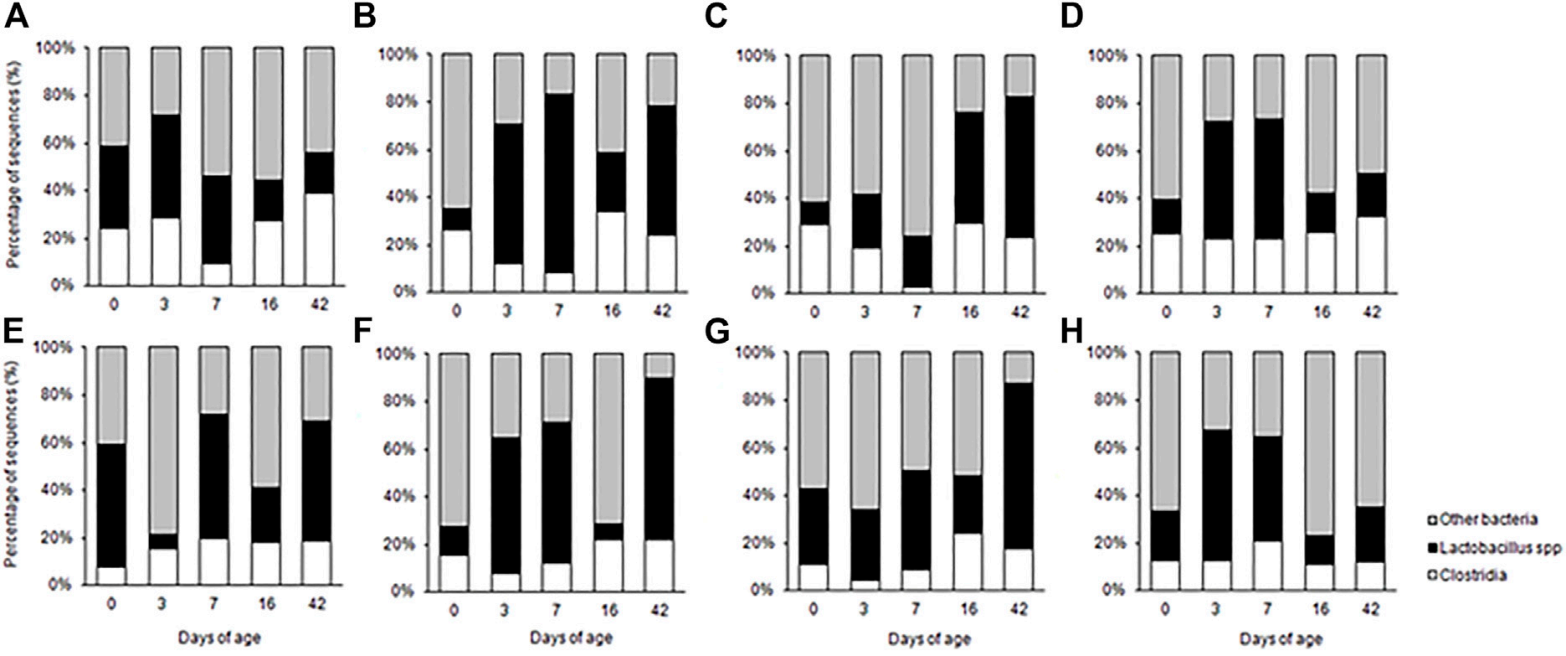
Composition of the small intestine bacterial community of chicks administered pioneer colonizers on day of hatch as determined by 16 S rRNA T-RFLP from samples collected from the jejunum **(A–D)** and ileum **(E–H)** of chickens from the *phosphate buffered saline* control group **(A, E)**, *Romboutsia lituseburense* and *Escherichia coli* cocktail **(B, F)**, *Parabacteroides distasonis*, *Bacteroides salyersiae*, *Phocaeicola dorei*, and *Escherichia coli* cocktail **(C, G)**, *P. distasonis*, *B. salyersiae*, *P. dorei*, *R. lituseburense* and *E. coli* cocktail **(D, H)**.

The probiotic cocktails were shown to modify the intestinal microbiota of birds compared to PBS control (Figure 4). Similar to Aviguard^®^ treatment, the probiotic formulations affected the *Lactobacillus* population in the intestine. With the exception of day 3, *P. distasonis*, *B. salyersiae*, *P. dorei*, *R. lituseburensis and E. coli* cocktail reduced ileal *Lactobacillus* abundance 23%–60% compared to the PBS control. However, other probiotic formulations increased *Lactobacillus* abundance, depending on the intestinal segment (jejunum vs. ileum) or day of age.

## Discussion

Poultry feed has diversified to vegetarian options and use of non-traditional ingredients that result in additional supplementation with amino acids and vitamins that enhance animal growth, physiology and performance (Alagawany et al., 2020). Gone are antibiotics once used to promote animal growth and prevent disease; replaced by probiotics, prebiotics, organic acids or essential oils. Some of these same feed additives have been shown to be comparable to growth-promoting antibiotics in improving intestinal development, animal growth, and pathogen exclusion or control (Gadde et al., 2017; Ricke, 2021; Abd El-Hack et al., 2022). These additives have been shown to alter the chicken gastrointestinal microbiome (Dittoe et al., 2018; Khan et al., 2020; Ali et al., 2021). The challenge now is piecing out their mechanism of action.

Poultry producers seek to imprint desirable attributes such as optimal feed conversion, disease and pathogen resistance, onto recipient hatchlings. Many studies have shown that a complex microbiota prohibits the establishment of harmful pathogens and fosters beneficial microbes that reduce inflammation, promote healing, improve feed efficiency and promote growth (van der Waaij et al., 1972; Candela et al., 2008; Fukuda et al., 2011; McNulty et al., 2011). Based on this concept, early intestinal colonization is essential to intestinal development, feed conversion and animal growth. Pioneer colonizers, as probiotics, offer an approach to ensure a mature and stable microbiome for newly hatched chicks.

Aviguard^®^, a commercially available competitive exclusion product, has been shown in multiple studies to improve disease resistance in broilers (Hofacre et al., 1998a; Hofacre et al., 1998b; Hofacre et al., 2019). In our current study it also altered the microbiome of chicks and improved development of the small intestine. The *Lactobacillus* population of the jejunum and ileum was more quickly replaced with intestinal anaerobes and the diversity of the ileal community was more stable indicating that the previously reported community successions could be altered (Lu et al., 2003). A more stable intestinal community structure in chicks at 3 weeks of age is important because this is a critical time of vulnerability for intestinal health (Hofacre et al., 1998a). Compositionally, the competitive exclusion product contained abundant intestinal member species, as potential pioneer colonizers, with sufficient diversity to induce intestinal development and animal growth (Flint et al., 2015; Kettle et al., 2015; Pedroso et al., 2015).

The most abundant bacterial phyla in the small intestine, following administration of the competitive exclusion product Aviguard^®^, were the phyla *Bacteroidetes* and *Firmicutes*, and specifically, with regard to the latter phyla, *Clostridia* was the dominant order. The impact of the intestinal microbiota on host physiology is being intensively studied and it is becoming increasingly clear that the intestine does not function or develop properly in the absence of its resident microbiota (Dubos et al., 1968; Smith et al., 2007; Sommer and Backhed, 2013). While *Clostridia* and *Bacteroidia* have fundamental differences in polysaccharide utilization and feeding strategies, they are similar in their reliance on carbohydrates for metabolism. *Bacteroidetes* harvest mucus glycans, a nutrient generated by its animal host (Koropatkin et al., 2012) but *Clostridia* are also known for their ability to harvest energy from indigestible fiber. The *Bacteroidia* and *Clostridia* species, examined in this study possessed many CAZymes for liberating sugars from mucin and indigestible fibers. In addition, the *Bacteroidia* have been shown to influence the carbohydrate composition of the intestinal glycome by liberating fucose by hydrolysis of mucin and the byproducts of fucose fermentation stimulate stem cell development (Bry et al., 1996). Fucose has been shown to be a terminal carbohydrate in the chicken’s intestinal glycome indicating that *Bacteroides* may also function as a pioneer colonizer in birds (Alroy et al., 1989; Madrid et al., 1989; Bryk et al., 1999). In addition, the species used in this study, *B. salyersiae*, *P. dorei*, and *R. lituseburensis,* possess fermentation enzymes and pathways for producing butyrate. While *P. distasonis* lacked these enzymes, it did possess enzymes necessary for producing propionate and several of the isolates also had acetyl-CoA hydrolases involved in acetate production.

Therefore, these probiotic isolates produce volatile fatty acids (VFA) that can be metabolized by the host animal. Metabolically, members of the order *Clostridia* and *Bacteroidia* cooperate rather than compete for the same nutrients in the intestine (Mahowald et al., 2009). This cooperation has the added benefit of increasing the VFA butyrate, which benefits their animal host in a number of ways including stimulating stem cell differentiation and reducing expression of inflammatory cytokines (Mahowald et al., 2009). Both *Clostridia* and *Bacteroidia* produce a variety of VFA, as end-products of fermentation, that can alter the composition of the microbiome and affect intestinal physiology (Segain et al., 2000; Pryde et al., 2002; Atarashi et al., 2013; Cockburn et al., 2015). Butyrate stimulates butyrate transporters in the host intestinal cells (Mahowald et al., 2009), dampens inflammation (Vieira et al., 2012), promotes intestinal integrity (Peng et al., 2009) and healing of intestinal damage (Butzner et al., 1996). In contrast, use of proteobacteria such as *E. coli* and *Citrobacter*, as pioneer colonizers in chicks elicited an intestinal inflammatory state that may lead to reduced intestinal health (Wilson et al., 2020; Chasser et al., 2021a; Chasser et al., 2021b).

The *P. distasonis* isolate used in this study also possessed a Vitamin B12 dependent ethanolamine utilization locus and vitamin B12 transporters that would allow it to compete with proteobacteria such as *Salmonella* and other intestinal bacteria for ethanolamine (Thiennimitr et al., 2011; Anderson et al., 2015; Kaval and Garsin, 2018). Furthermore, these *Clostridia* and *Bacteroidia* species may have elicited an indirect improvement of feed conversion by suppression of the *Lactobacillus* population. The lactobacilli are auxotrophs, deficient in their ability to synthesize up to eight different amino acids, vitamins and important co-factors (Makarova et al., 2006; Cai et al., 2009). While they are capable of fermenting a large repertoire of carbohydrates, they do not possess the enzymes to acquire these sugars from mucin (Makarova et al., 2006; Cai et al., 2009). Therefore, *Lactobacillus* is in competition with its host for free sugars, peptides and amino acids while the strict anaerobes such as *Clostridia* and *Bacteroidia* focus on utilizing mucin. Under feed restriction or a diet with low digestibility such as a wheat vs corn-soy diet, the composition of the small intestinal microbiome may have a significant impact on feed conversion and weight gain because of this competition (Torok et al., 2008; Metzler-Zebeli et al., 2019). In fact, a negative correlation between *Lactobacillus* abundance in the ileum and total body weight gain has been shown under feed restriction (Metzler-Zebeli et al., 2019). Low body weight birds tend to also have microbiome dominated by lactic acid bacteria (Zhao et al., 2013).

This is not to say the lactobacilli do not perform important functions for its animal host including dampening inflammation (Chen et al., 2012; Gong et al., 2020; Šefcová et al., 2020) or pathogen exclusion (Chen et al., 2012; Gong et al., 2020). However the mechanism of action of growth-promoting antibiotics may not only be due to suppression of pathogens (Arakawa and Oe, 1975), but the streptogramin (Lamb et al., 1999), glycopeptide (Chow and Cheng, 1988) and bacitracin (Toscano and Storm, 1982) antibiotics have broad activity against lactobacilli. In fact, antibiotic growth promoters suppress *Lactobacillus*, reduce community diversity and favor *Clostridia* in the ileum, similar to results observed with the competitive exclusion product used in this study (Lu et al., 2008). Therefore it is not surprising that growth promoting antibiotics profoundly affect microbiome composition and diversity (Lu et al., 2008). And while the growth-promoting properties of antibiotics and probiotics might be attributed to control of intestinal pathogens such as *C. perfringens*, it is also likely that their true impact is from enhancing intestinal development, modulating metabolism of the microbiome, and allowing the animal to better compete for nutrients liberated in the small intestine.

The *Bacteroidia* contain foundational genera, *Bacteroides* and *Parabacteroides* transmitted from the adult hen to its progeny, when hens are reared with their chicks (Kubasova et al., 2019a). The *Bacteroidetes* become the dominant phyla by day 18, for gnotobiotic chicks seeded with the intestinal microbiome from feral chickens (Thomas et al., 2019) and members of this phyla can stably colonize the cecum of chicks administered a complex cocktail containing this phyla, *Firmicutes* and *Proteobacteria* (Kubasova et al., 2019b). *Bacteroidia* member species have been shown to exclude certain pathogens from the chicken gastrointestinal tract (Kubasova et al., 2019b; Papouskova et al., 2023).

While we observed significant improvement to intestinal development and animal performance with our five-member probiotic formulation, this does not imply that this probiotic performs all the same functions as the competitive exclusion product examined in this study. While variable in composition, this product is consistent at reducing *Salmonella* colonization in chicks (Lee et al., 2023) and has been shown to be effective at controlling other enteropathogens (Hofacre et al., 1998a; Hofacre et al., 2019). This competitive exclusion product is a complex consortium, of chicken cecal origin, that consists of 20–50 distinct genera. While Kubasova et al. demonstrated significant *Salmonella* exclusion with an eight-member probiotic formulation, including *P. distasonis* (Kubasova et al., 2019b), it is not known whether this same formulation can exclude other enteropathogens or has growth promoting properties. Perhaps, it requires sufficient community diversity to outcompete pathogens, promote intestinal development and function, and repair any perturbation to gut function brought about by disease. Microbiome diversity is important in pathogen exclusion (Pedroso et al., 2021) and restoring homeostasis following any perturbation to the gastrointestinal tract (Weimer, 2015).

## Conclusion

In addition to excluding pathogens, competitive exclusion product contains foundational bacterial species to promote intestinal function, development and animal growth. Intestinal pioneering colonizers selected from chicken ceca, based on their prominence in competitive exclusion product and consisting of five-member intestinal species, was comparable to a competitive exclusion product in improving intestinal morphology and animal performance. The balance of proteobacter, lactobacilli and anaerobic intestinal member species is critical to a healthy microbiome that promotes intestinal development, feed efficiency and animal growth (Foo et al., 2017). In the past, growth-promoting antibiotics provided this balance. Now, as the poultry industry has moved towards antibiotic free production, defined intestinal bioproducts are needed to stimulate intestinal development and function, support lower feed conversion rates and improved body weight gains, and maintain a healthy balance in the intestinal microbiota.

## Data Availability

The original contributions presented in the study are included in the article/Supplementary Materials, further inquiries can be directed to the corresponding author.
