# Supplementary figures and images for: Pioneer colonizers: Bacteria that alter the chicken intestinal morphology and development of the microbiota
Source: Front Physiol. 2023 Mar 29;14:1139321. doi: 10.3389/fphys.2023.1139321 (PMC10090334; doi:10.3389/fphys.2023.1139321)

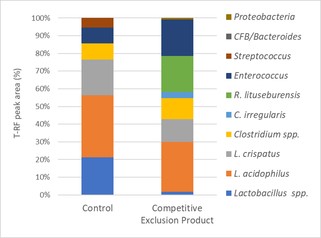

Supplement: Supplementary file 1 [file Image1.JPEG]

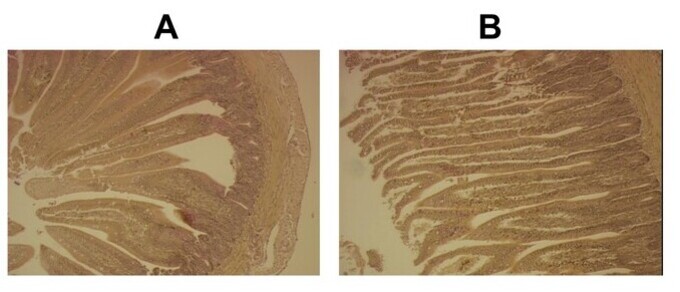

Supplement: Supplementary file 2 [file Image2.JPEG]
