# Supplementary material for: Pioneer colonizers: Bacteria that alter the chicken intestinal morphology and development of the microbiota
Source: Front Physiol. 2023 Mar 29;14:1139321. doi: 10.3389/fphys.2023.1139321 (PMC10090334; doi:10.3389/fphys.2023.1139321)
Supplement: Supplementary file 3 [file DataSheet1.docx]

**Table S1**. Composition of the diets (as fed basis)

| Ingredients | Starter | Grower | Finisher |
| --- | --- | --- | --- |
| Corn | 56.128 | 60.793 | 68.015 |
| Soybean meal (48) | 37.500 | 32.614 | 26.220 |
| Poultry fat | 3.000 | 3.433 | 2.990 |
| Dicalcium phosphate | 1.751 | 1.563 | 1.315 |
| Limestone | 0.800 | 0.780 | 0.620 |
| Salt | 0.295 | 0.320 | 0.350 |
| Vitamin premix | 0.250 | 0.250 | 0.250 |
| DL-Methionine | 0.201 | 0.172 | 0.165 |
| Trace mineral premix | 0.075 | 0.075 | 0.075 |
| Contents by calculation |  |  |  |
| ME, kcal/kg | 3095 | 3140 | 3191 |
| Protein, % | 22.6 | 20.6 | 18.1 |
| Lysine, % | 1.14 | 1.01 | 0.85 |
| Methionine, % | 0.53 | 0.48 | 0.45 |
| TSAA, % | 0.84 | 0.76 | 0.70 |

^1^Vitamin mix provided the following (per kg of diet): thiamin •mononitrate, 2.4 mg; nicotinic acid, 44 mg; riboflavin, 4.4 mg; D-Ca pantothenate, 12 mg; vitamin B_12_ (cobalamin),12.0 µg; pyridoxine •HCL, 4.7 mg; D-biotin, 0.11 mg; folic acid, 5.5 mg; menadione sodium bisulfite complex, 3.34 mg; choline chloride, 220 mg; cholecalciferol, 27.5 ug; trans-retinyl acetate, 1,892 ug; all-rac α tocopheryl acetate, 11 mg; ethoxyquin, 125 mg.

^2^Trace mineral mix provided the following (per kg of diet): manganese (MnSO_4_•H_2_O), 60 mg; iron (FeSO_4_•7H_2_O), 30 mg; zinc (ZnO), 50 mg; copper (CuSO_4_•5H_2_O), 5 mg; iodine (ethylene diamine dihydroiodide), 0.15 mg; selenium (NaSe0_3_), 0.3 mg.

**Table S2**. Relative intestinal weight and length of the ileum of chickens administered *Bacteroidia* (Parabacteroides distasonis, *Bacteroides salyersiae*, and *Phocaeicola dorei*) (Bac), *Romboutsia lituseburensis* (Clos) or a mixture of *Bacteroidia* and *R. lituseburensis* (Bac + Clos).

|  | 0d | 1d | 2d | 3d | 7d | 16d | 42d |
| --- | --- | --- | --- | --- | --- | --- | --- |
| Treatment | Relative weight (g/kg of body weight) | | | | | | |
| Control | 7.8 | 14.3 | 17.1 | 19.9 | 19.6 | 15.7 | 8.0 |
| *Bac* | 11.7 | 13.7 | 17.6 | 20.1 | 20.3 | 16.9 | 7.2 |
| *Clos* | 7.4 | 15.1 | 17.5 | 22.7 | 19.5 | 16.0 | 8.0 |
| *Bac + Clos* | 8.8 | 14.2 | 17.7 | 22.4 | 19.5 | 15.0 | 7.3 |
| Pooled SEM | 1.20 | 0.76 | 0.84 | 1.07 | 0.76 | 0.58 | 0.45 |
|  | Relative length (cm/kg of body weight) | | | | | | |
| Control | 269 | 302 | 286 | 304 | 179 | 91 | 27 |
| *Bac* | 315 | 312 | 286 | 286 | 183 | 86 | 28 |
| *Clos* | 294 | 321 | 309 | 314 | 193 | 87 | 29 |
| *Bac + Clos* | 280 | 296 | 315 | 303 | 180 | 87 | 28 |
| Pooled SEM | 14.53 | 14.95 | 9.67 | 12.20 | 5.85 | 2.87 | 0.99 |

^a-c^ Means within a column with no common superscript differ significantly (P < 0.05).
